# Comparison of preoperative NT-proBNP and simple cardiac risk scores for predicting postoperative morbidity after non-cardiac surgery with intermediate or high surgical risk

**DOI:** 10.1186/s13741-024-00400-z

**Published:** 2024-05-17

**Authors:** Götz Schmidt, Nora Frieling, Emmanuel Schneck, Marit Habicher, Christian Koch, Birgit Aßmus, Michael Sander

**Affiliations:** 1 Department of Anaesthesiology, Intensive Care Medicine and Pain Therapy, Justus Liebig University Giessen, Rudolf-Buchheim-Strasse 7, Giessen, 35392 Germany; 2 Department of Cardiology and Angiology, Justus Liebig University of Giessen, Klinikstrasse 33, Giessen, 35392 Germany

**Keywords:** Revised cardiac risk index, AUB-HAS2, Brain natriuretic peptide, Perioperative, Rehospitalisation, Acute kidney injury, Acute decompensated heart failure

## Abstract

**Background:**

Chronic heart failure (HF) is frequent in elderly patients undergoing non-cardiac surgery. Preoperative risk stratification is vital and can be achieved using simple clinical risk scores or preoperative N-terminal prohormone of brain natriuretic peptide (NT-proBNP) measurement. This study aimed to compare the predictivity of the revised cardiac risk index (RCRI), the American University of Beirut cardiovascular risk index (AUB-HAS2), and a score proposed by Andersson et al. for postoperative 30-day morbidity to preoperative NT-proBNP.

**Methods:**

Preoperative NT-proBNP was measured in 199 consecutive patients aged ≥ 65 years undergoing elective non-cardiac surgery with intermediate or high surgical risk. The areas under the receiver operating characteristic curve (AUCROC) for the composite morbidity endpoint (CME) comprising the incidence of any rehospitalisation, acute decompensated HF, acute kidney injury, and any infection at postoperative day 30 were assessed. Multivariable logistic regression analysis derived new scores from the simple risk scores and the NT-proBNP cut-off of 450 pg/mL.

**Results:**

AUB-HAS2, but not RCRI or Andersson score, significantly predicted the CME (AUB-HAS2: AUCROC 0.646, *p* < 0.001; RCRI: AUCROC 0.560,* p* = 0.126; Andersson: AUCROC 0.487, *p* = 0.760). The AUCROC was comparable between preoperative NT-proBNP (0.679, *p* < 0.001) and AUB-HAS2 (*p* = 0.334). Multivariable analyses revealed a preoperative NT-proBNP ≥ 450 pg/mL to be the strongest predictor of CME among the individual score components (*p* < 0.001). Adding preoperative NT-proBNP improved the predictive value of AUB-HAS2 and RCRI (modified AUB-HAS2: AUCROC 0.703, *p* < 0.001; modified RCRI: AUCROC 0.679, *p* < 0.001; both *p* < 0.001 vs original scores). The predictive value of the modified RCRI and AUB-HAS2 was comparable to preoperative NT-proBNP alone (*p* = 0.988 vs modified RCRI, *p* = 0.367 vs modified AUB-HAS2).

**Conclusions:**

The predictive value of postoperative morbidity varies significantly between the available simple perioperative risk scores and can be enhanced by preoperative NT-proBNP. New scores, including preoperative NT-proBNP, should be evaluated in large multicentre cohorts.

**Trial registration:**

German Clinical Trials Register: DRKS00027871.

**Supplementary Information:**

The online version contains supplementary material available at 10.1186/s13741-024-00400-z.

## Background

Chronic heart failure (HF) is frequent in ageing populations and is associated with reduced quality of life and increased morbidity and mortality (McDonagh et al. 2021; Gerber et al. 2015; Shah et al. 2017). The general prevalence of chronic HF is estimated at 1 to 2% but is markedly higher in populations aged > 65 years, where it affects 10% of patients (Groenewegen et al. 2020). Therefore, elderly patients requiring major non-cardiac surgery are at particular risk of cardiovascular complications, such as acute decompensated HF (ADHF), infections, acute kidney injury (AKI), and death (Farzi et al. 2013; Schmidt et al. 2024). A recent prospective observational study reported a 2.5% rate of postoperative ADHF among 9164 patients. One striking finding of this study was that 51% of postoperative ADHF occurred in patients without a known history of HF (Gualandro et al. 2023). Therefore, risk stratification and detection of potential unknown HF and congestion are critical in the preoperative setting to apply risk-mitigating strategies to prevent postoperative complications in this vulnerable population. However, with standard tools, detecting and assessing unknown but compensated HF only via physical examination in the preoperative anaesthesiologic visit might be challenging, if not impossible.

Therefore, current European Society of Cardiology (ESC) guidelines on cardiovascular assessment and management in patients undergoing non-cardiac surgery recommend obtaining an accurate patient history, including a focused clinical examination; however, further risk stratification measures are also proposed (Halvorsen et al. 2022). For example, the 30-day risk for cardiovascular death, stroke, and myocardial infarction can be estimated solely from the surgery type, which is 1–5% and > 5% for surgeries with intermediate or high risk, respectively. In the 2022 ESC guidelines, measuring cardiac biomarkers in patients aged > 65 years, such as high-sensitivity cardiac troponin T/I and brain natriuretic peptides (BNP) or its precursor N-terminal prohormone of BNP (NT-proBNP), has emerged as a class I or II recommendation, respectively. Since NT-proBNP showed good predictivity and risk stratification in chronic and acute HF populations, it might be particularly suited to identify patients with HF, which could then be preoperatively optimised (McDonagh et al. 2021).

Moreover, patients with chronic HF undergoing non-cardiac surgery showed much higher mortality and hospital readmission rates at 30 days than patients without HF or with chronic coronary syndrome (Halvorsen et al. 2022; Lee et al. 1999). Furthermore, current guidelines state that clinical risk scores, most of which include both patient-related and surgery-related risk factors, can also be considered when assessing the perioperative risk of an elderly patient undergoing major non-cardiac surgery (Halvorsen et al. 2022). However, the current guidelines do not recommend a specific risk score, because none can be regarded as a gold standard compared to others based on current evidence (Halvorsen et al. 2022).

Simple risk scores are calculated by summing a numeric value for each score criterion met. For example, the revised cardiac risk index (RCRI) was introduced in 1999 and is calculated from the presence of chronic HF, coronary artery disease, cerebrovascular disease, insulin therapy, preoperative serum creatinine > 2 mg/dL, and high-risk surgery type, which is defined as intraperitoneal, intrathoracic, or suprainguinal vascular surgery (Lee et al. 1999). The RCRI has been validated in various studies and showed moderate predictivity for the risk of cardiac death, myocardial infarction, and nonfatal cardiac arrest in patients undergoing non-cardiac surgery (Ford et al. 2010).

Andersson et al. proposed a new risk score in 2014 to predict 30-day mortality in patients with HF undergoing non-cardiac surgery, which included sex, age, body mass index, acute surgery, insulin therapy, renal disease, cerebrovascular disease, and high-risk procedure, which was defined according to the RCRI definition (Andersson et al. 2014). In 2019, Dakik et al. introduced the American University of Beirut Cardiovascular Risk Index (AUB-HAS2), which is calculated based on age ≥ 75 years; history of heart disease, angina, or dyspnoea; haemoglobin < 12 g/dL; emergency surgery; and vascular surgery (Dakik et al. 2019a, 2019b). The primary outcome measure of the AUB-HAS2 is death, myocardial infarction, or stroke at 30 days.

In summary, while these three simple clinical risk scores all aim to predict perioperative outcomes, they mainly focus on myocardial infarction, cardiac arrest, and death (Halvorsen et al. 2022). None of the currently available clinical risk scores considers preoperative NT-proBNP testing for risk prediction, and none has been validated for clinically relevant postoperative morbidity measures, such as ADHF, AKI, and infections. Therefore, this study aimed to compare the predictive value of the RCRI, AUB-HAS2, and Andersson scores with preoperative NT-proBNP for the postoperative 30-day morbidity in an observational non-cardiac and non-vascular surgery cohort. Additionally, we assessed whether the predictivity of these scores could be improved by adding a preoperative NT-proBNP cut-off into their respective score.

## Methods

### Study design

This study was a secondary analysis of a prospective, single-centre cohort study that aimed to evaluate patients’ postoperative morbidity stratified by preoperative NT-proBNP after non-cardiac and non-vascular surgery with intermediate or high surgical risk (Schmidt et al. 2024). The study was approved by the local ethics committee of the Medical Faculty of Justus Liebig University, Giessen, Germany (approval number: AZ 263/21), and was performed according to the Declaration of Helsinki. Given its observational character and data anonymisation, written informed consent to participate was waived. This study was registered with the German Clinical Trials Register (ID: DRKS00027871; registration date: 17 January 2022). It prospectively enrolled 200 consecutive patients aged > 65 years scheduled for elective non-cardiac non-vascular surgery in general anaesthesia with intermediate or high surgical risk according to the current ESC guidelines on cardiovascular assessment and management in patients undergoing non-cardiac surgery (Halvorsen et al. 2022). The inclusion criteria were elective intracranial, thoracic, head and neck, trauma and orthopaedic, or abdominal surgery, including visceral, urological, and gynaecological operations. Consequently, patients aged < 65 years, undergoing cardiac or vascular surgery, or undergoing surgery with regional anaesthesia and emergency patients were excluded. Before the presurgical anaesthetic visit, NT-proBNP was measured in every patient using venous blood with a point-of-care immunoassay (proBNP + , cobas h 232; Roche Holding AG, Basel, Switzerland). Anaesthetic management and intra- and post-operative treatment were performed according to local institutional standards in compliance with the current guidelines.

### Data acquisition

Data were collected during the routine presurgical visit at the anaesthesia outpatient clinic. Baseline characteristics included age, sex, cardiovascular risk factors, history of chronic HF or myocardial infarction, and relevant comorbidities, such as peripheral artery disease, carotid artery stenosis, chronic obstructive pulmonary disease, pulmonary hypertension, stroke, and chronic kidney disease. These preoperatively recorded baseline characteristics were used to calculate the RCRI, AUB-HAS2, and Andersson scores.

### Endpoints

The primary endpoint of the study was the composite morbidity endpoint (CME), comprising the incidence of any rehospitalisation, ADHF, AKI, and any suspected or proven bacterial infection requiring treatment after surgery until POD 30. Event rates were compared between patients with preoperative NT-proBNP ≥ 450 pg/mL and < 450 pg/mL. The 450 pg/mL cut-off is described in detail elsewhere (Schmidt et al. 2024). AKI was defined according to the Kidney Disease: Improving Global Outcomes criteria, and ADHF was defined as the onset or worsening of shortness of breath and signs of congestion, including peripheral oedema, moist rales, and radiological signs of congestion or pleural effusion, requiring treatment (Walther et al. 1995). The predictive value of the RCRI, AUB-HAS2, and Andersson scores was evaluated in this secondary analysis.

### Statistical analysis

The RCRI, AUB-HAS2, and Andersson scores and their components were calculated and compared between patients stratified by the clinically implemented preoperative NT-proBNP cut-off. Categorial variables are presented as numbers and percentages and were compared between groups using the chi-squared or Fisher’s exact test. Continuous variables are presented as medians and interquartile ranges and were compared between groups using the Mann–Whitney-Wilcoxon test. The correlations between preoperative NT-proBNP values and RCRI, AUB-HAS2, and Andersson scores were assessed using Spearman’s rank correlation coefficient (r_s_) and classified as very weak (0 < *r*_s_ < 0.2), weak (0.2 < *r*_s_ < 0.4), moderate (0.4 < *r*_s_ < 0.6), strong (0.6 < *r*_s_ < 0.8), and very strong (*r*_s_ ≥ 0.8). Areas under the curve of the receiver operating characteristic (AUCROC) were calculated to determine the discriminatory power of preoperative NT-proBNP, RCRI, AUB-HAS2, and Andersson scores in predicting the CME and were compared using a *z*-test. Each optimal discriminatory level was identified according to Youden’s index, and cut-offs are reported with their sensitivity and specificity. Multivariable logistic regression analysis was performed for the individual score components to identify independent predictors of the CME and assess their value in combination with the preoperative NT-proBNP cut-off. New scores were then derived from the simple risk scores using their components and preoperative NT-proBNP, where all components were weighted by their calculated odds ratios in the multivariable logistic regression models. Two-tailed *p*-values < 0.05 were considered statistically significant. Statistical analyses were performed using IBM SPSS Statistics (version 28.0.0.1; IBM, Armonk, NY, USA).

## Results

### Baseline characteristics and scores

Baseline characteristics, including the RCRI, AUB-HAS2, and Andersson scores and their components, are shown in Table 1. Patients with preoperative NT-proBNP ≥ 450 pg/mL were significantly older than those with preoperative NT-proBNP < 450 pg/mL and had higher median RCRI and AUB-HAS2 scores (all *p* < 0.001). However, Andersson scores were comparable between the two groups. Among the RCRI components, a history of chronic HF and coronary artery disease, preoperative insulin therapy, and serum creatinine > 2 mg/dL were more frequently present in patients with preoperative NT-proBNP ≥ 450 pg/mL. All AUB-HAS2 components were more frequently observed in patients with preoperative NT-proBNP ≥ 450 pg/mL. Since our study did not include patients who underwent emergency or vascular surgery, these criteria could not be met in this analysis. In contrast, despite the prevalence of preoperative insulin therapy, all Andersson risk score components were comparable between the two groups.

**Table 1:** Baseline characteristics and scores

Characteristics	Total (*n* = 199)	NT-proBNP ≤ 450 pg/mL (*n* = 129)	NT-proBNP > 450 pg/mL (*n* = 70)	*p*
Patient characteristics				
Median age [IQR] — years	74 [69–81]	72 [68–77]	80 [74–83]	< *0.001*
NT-proBNP [IQR] — pg/mL	277 [134–779]	173 [103–274]	1309 [730–2918]	< *0.001*
Revised cardiac risk index				
Median revised cardiac risk index [IQR] — no	1 [0–2]	1 [0–1]	1[1–2]	< *0.001*
High-risk type of surgery — no. (%)	77 (38.7)	54 (41.9)	23 (32.9)	*0.274*
Chronic heart failure — no. (%)	21 (10.6)	4 (3.1)	17 (24.3)	< *0.001*
Coronary artery disease — no. (%)	51 (25.6)	22 (17.1)	29 (25.6)	< *0.001*
Cerebrovascular disease — no. (%)	21 (10.6)	10 (7.8)	11 (15.7)	*0.133*
Insulin therapy — no. (%)	23 (11.6)	10 (7.8)	13 (18.6)	*0.041*
Serum creatinine > 2 mg/dL — no (%)	13 (6.5)	1 (0.8)	12 (17.1)	< *0.001*
American University of Beirut Cardiovascular Risk Index				
Median AUB-HAS2 score [IQR] — no	1 [0–2]	1 [0–1]	2 [2–3]	< *0.001*
Age ≥ 75 years — no. (%)	92 (46.2)	42 (32.6)	50 (71.4)	< *0.001*
History of heart disease — no. (%)	77 (38.7)	27 (20.9)	50 (71.4)	< *0.001*
Angina or dyspnoea — no. (%)	31 (15.6)	10 (7.8)	21 (30.0)	< *0.001*
Haemoglobin < 12 g/dL — no. (%)	68 (34.2)	25 (19.4)	43 (61.4)	< *0.001*
Emergency surgery — no. (%)	0 (0.0)	0 (0.0)	0 (0.0)	1.000
Vascular surgery — no. (%)	0 (0.0)	0 (0.0)	0 (0.0)	1.000
Andersson et al				
Median risk score [IQR] — no	9 [7–10]	9 [7–10]	8 [7–10]	0.777
Male — no. (%)	101 (50.8)	63 (48.8)	38 (54.3)	0.463
Age				
Age 56–65 years — no. (%)	7 (3.5)	4 (3.1)	3 (4.3)	0.698
Age 66–75 years — no. (%)	110 (55.3)	70 (54.3)	40 (57.1)	0.810
Age 76–85 years — no. (%)	71 (35.7)	50 (38.8)	21 (30.0)	0.282
Age ≥ 85 years — no. (%)	11 (5.5)	5 (3.9)	6 (8.6)	0.289
Body mass index (BMI)				
Underweight — no. (%)	6 (3.0)	3 (2.3)	3 (4.3)	0.735
Normal weight — no. (%)	73 (36.7)	46 (35.7)	27 (38.6)	0.800
Overweight — no. (%)	80 (40.2)	52 (40.3)	28 (40.0)	1.000
Obese — no. (%)	40 (20.1)	28 (21.7)	12 (17.1)	0.561
Acute surgery — no. (%)	0 (0.0)	0 (0.0)	0 (0.0)	1.000
High-risk type of surgery — no. (%)	77 (38.7)	54 (41.9)	23 (32.9)	0.274
Renal disease — no. (%)	131 (65.8)	79 (61.2)	52 (74.3)	0.090
Cerebrovascular disease — no. (%)	21 (10.6)	10 (7.8)	11 (15.7)	0.133
Insulin therapy — no. (%)	23 (11.6)	10 (7.8)	13 (18.6)	0.041

Preoperative NT-proBNP values were very weakly correlated with Andersson scores (*r*_s_ = 0.176), weakly correlated with RCRI scores (*r*_s_ = 0.339), and strongly correlated with AUB-HAS2 scores (*r*_s_ = 0.635; Supplemental Fig. 1).

**Fig. 1:**
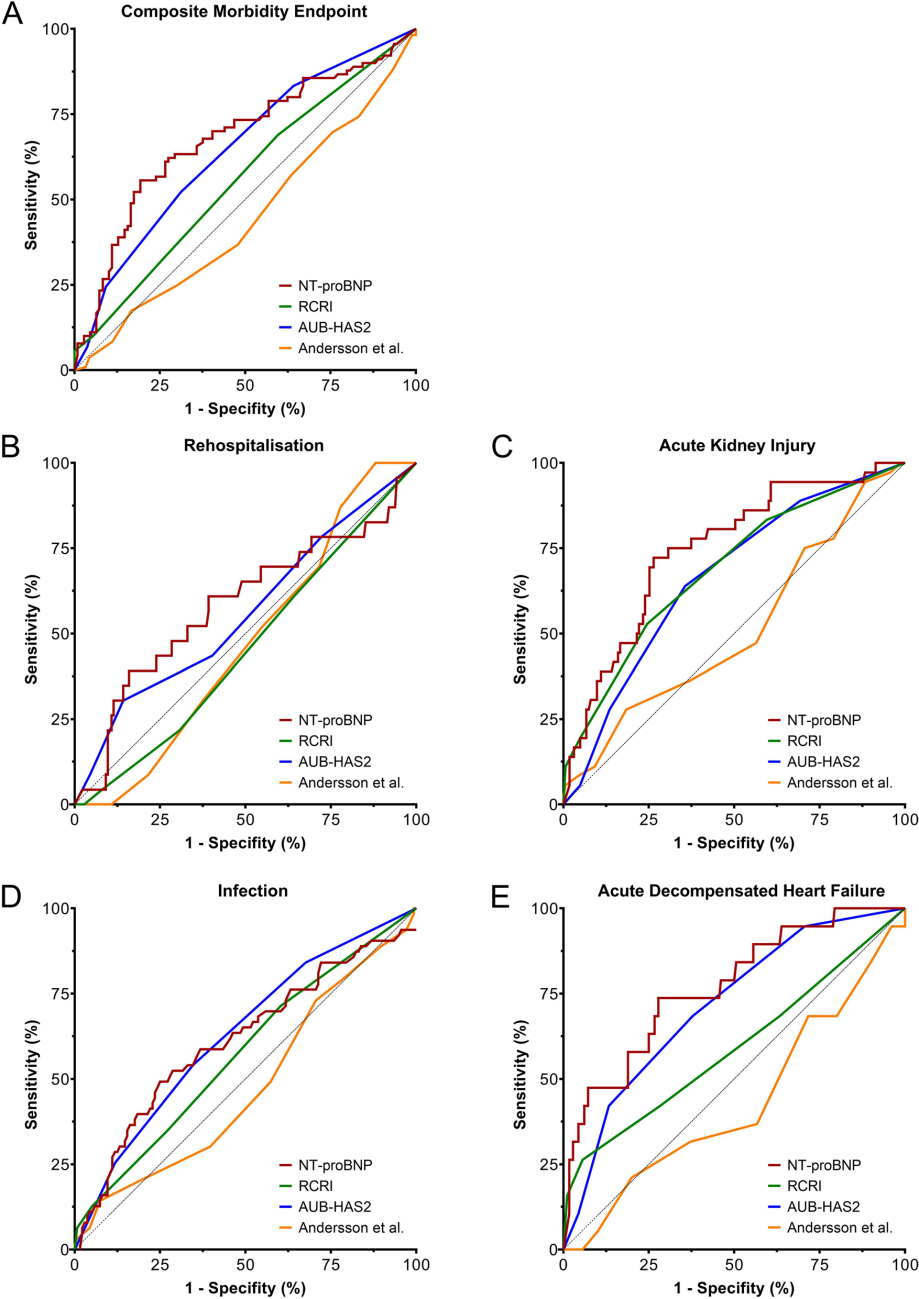
Predictive abilities of preoperative NT-proBNP and clinical risk scores. Receiver operating characteristic curves are shown for **A** the composite morbidity endpoint, comprising **B** rehospitalisation, **C** acute kidney injury, **D** infection, and **E** acute decompensated heart failure during the first 30 days after surgery. Abbreviations: RCRI, Revised cardiac risk index; AUB-HAS2, American University of Beirut Cardiovascular Risk Index

### Predictive value of clinical risk scores and preoperative NT-proBNP

Preoperative NT-proBNP and AUB-HAS2 could predict the CME, while the AUCROC for the RCRI and Andersson scores did not reach statistical significance. The AUCROC, calculated cut-offs, and their specificity and sensitivity in predicting the CME and the individual components are shown in Fig. 1 and Table 2. The AUCROC was significantly larger for preoperative NT-proBNP than for the RCRI (*p* = 0.008) and Andersson score (*p* = 0.001) but did not differ statistically between preoperative NT-proBNP and AUB-HAS2 (*p* = 0.334). When the individual CME components were analysed, all scores and preoperative NT-proBNP failed to predict any rehospitalisation within our study’s observation period. Furthermore, the Andersson score could not predict any individual CME component. However, RCRI could predict AKI but not infection and ADHF in our cohort. Preoperative NT-proBNP and AUB-HAS2 could predict AKI, infection, and ADHF, with the numerically largest AUCROC observed for preoperative NT-proBNP in predicting AKI and ADHF. The calculated optimal preoperative NT-proBNP cut-offs ranged from 443 pg/mL (CME) to 482 pg/mL (ADHF and infection), while RCRI and AUB-HAS2 scores > 2 showed the best discrimination for AKI, infection (AUB-HAS2 only), and ADHF. The highest sensitivity and specificity for CME, ADHF, and AKI were obtained with preoperative NT-proBNP, although the AUB-HAS2 showed the highest sensitivity in predicting postoperative infections. The AUCROC for AKI was significantly larger with preoperative NT-proBNP than with AUB-HAS2 (*p* = 0.023) but did not differ significantly between preoperative NT-proBNP and RCRI (*p* = 0.255). The AUCROC for ADHF (*p* = 0.337) and infections (*p* = 0.739) did not differ significantly between preoperative NT-proBNP and AUB-HAS2.

**Table 2:** The predictivity of preoperative NT-proBNP and the clinical risk scores for the composite morbidity endpoint and its components

Score	AUCROC	*p*	Cut-off	Sensitivity	Specificity
Composite morbidity endpoint					
NT-proBNP	0.679	< *0.001*	443 pg/mL	55.6%	80.7%
Revised Cardiac Risk Index	0.560	0.126			
AUB-HAS2 risk score	0.646	< *0.001*	2	52.2%	68.8%
Andersson et al. risk score	0.487	0.760			
Rehospitalisation					
NT-proBNP	0.585	0.236			
Revised Cardiac Risk Index	0.457	0.461			
AUB-HAS2 risk score	0.559	0.371			
Andersson et al. risk score	0.473	0.606			
Acute kidney injury					
NT-proBNP	0.747	< *0.001*	453 pg/mL	72.2%	73.6%
Revised Cardiac Risk Index	0.687	< *0.001*	2	52.8%	75.5%
AUB-HAS2 risk score	0.664	< *0.001*	2	63.9%	64.4%
Andersson et al. risk score	0.510	0.852			
Infection					
NT-proBNP	0.619	*0.008*	482 pg/mL	49.2%	75.7%
Revised Cardiac Risk Index	0.573	0.081			
AUB-HAS2 risk score	0.632	*0.001*	2	54.0%	65.4%
Andersson et al. risk score	0.478	0.623			
Acute decompensated heart failure					
NT-proBNP	0.769	< *0.001*	482 pg/mL	73.7%	72.2%
Revised Cardiac Risk Index	0.590	0.242			
AUB-HAS2 risk score	0.715	< *0.001*	2	68.4%	62.2%
Andersson et al. risk score	0.430	0.334			

Abbreviations: *AUCROC* area under the receiver operating characteristic curve, *AUBHAS2* American University of Beirut Cardiovascular Risk Index

### Independent predictors of postoperative morbidity

**Fig. 2:**
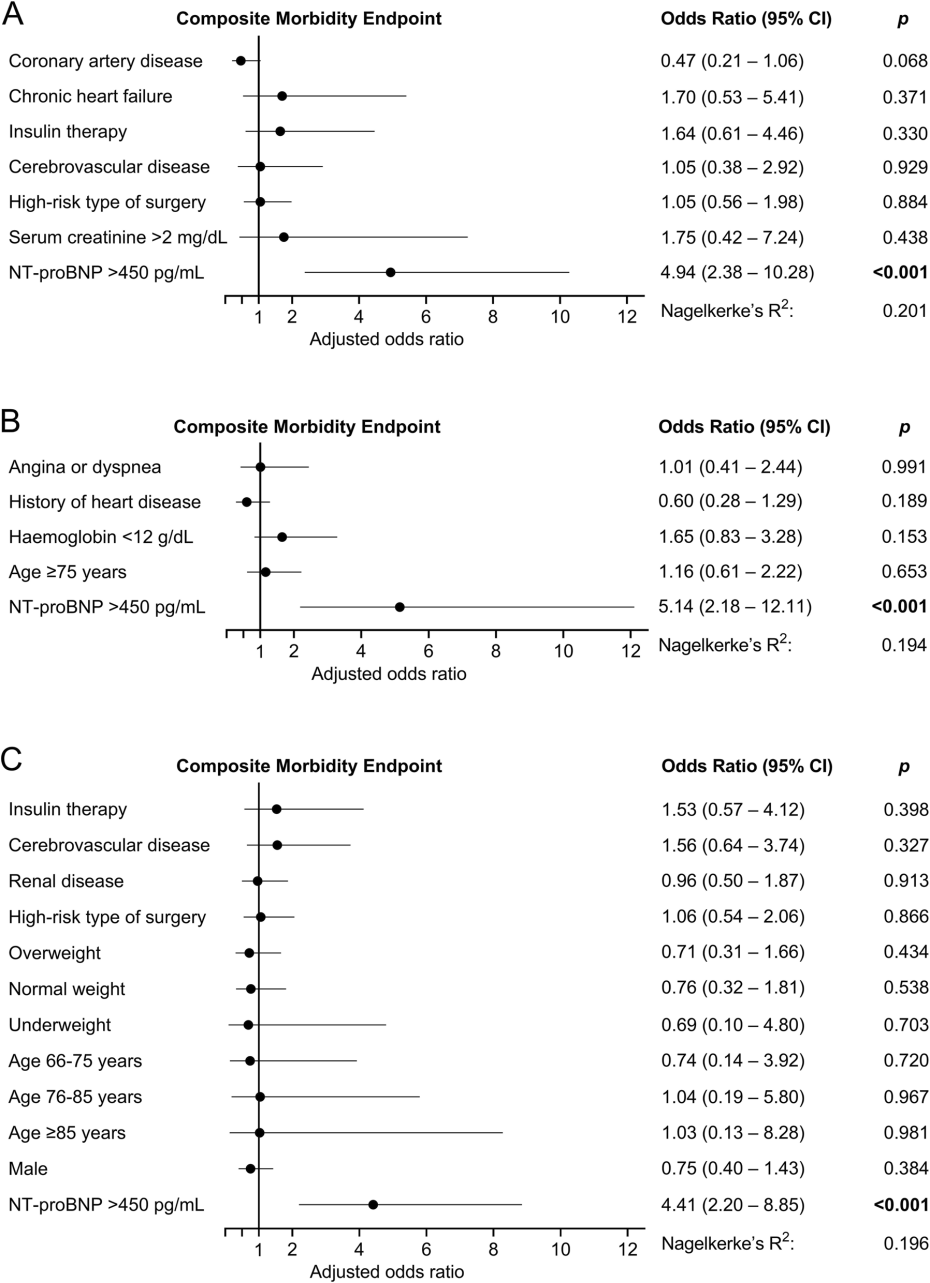
Multivariable logistic regression analyses combining clinical risk scores with preoperative NT-proBNP. When the preoperative NT-proBNP cut-off of 450 pg/mL was included, preoperative NT-proBNP was the only independent predictor of postoperative morbidity, outweighing the clinical parameters used in **A** the revised cardiac risk index (RCRI), **B** the American University of Beirut Cardiovascular Risk Index (AUB-HAS2), and **C** the Andersson score

Multivariable logistic regression models containing the individual score components and the clinically adjusted preoperative NT-proBNP cut-off of 450 pg/mL are shown in Fig. 2. Preoperative NT-proBNP > 450 pg/mL was the strongest independent predictor of CME in each examined model. Furthermore, the original individual score components did not independently predict the CME when the strong predictor preoperative NT-proBNP was added to the respective model.

### Improving predictivity of clinical risk scores by adding the preoperative NT-proBNP cut-off

**Fig. 3:**
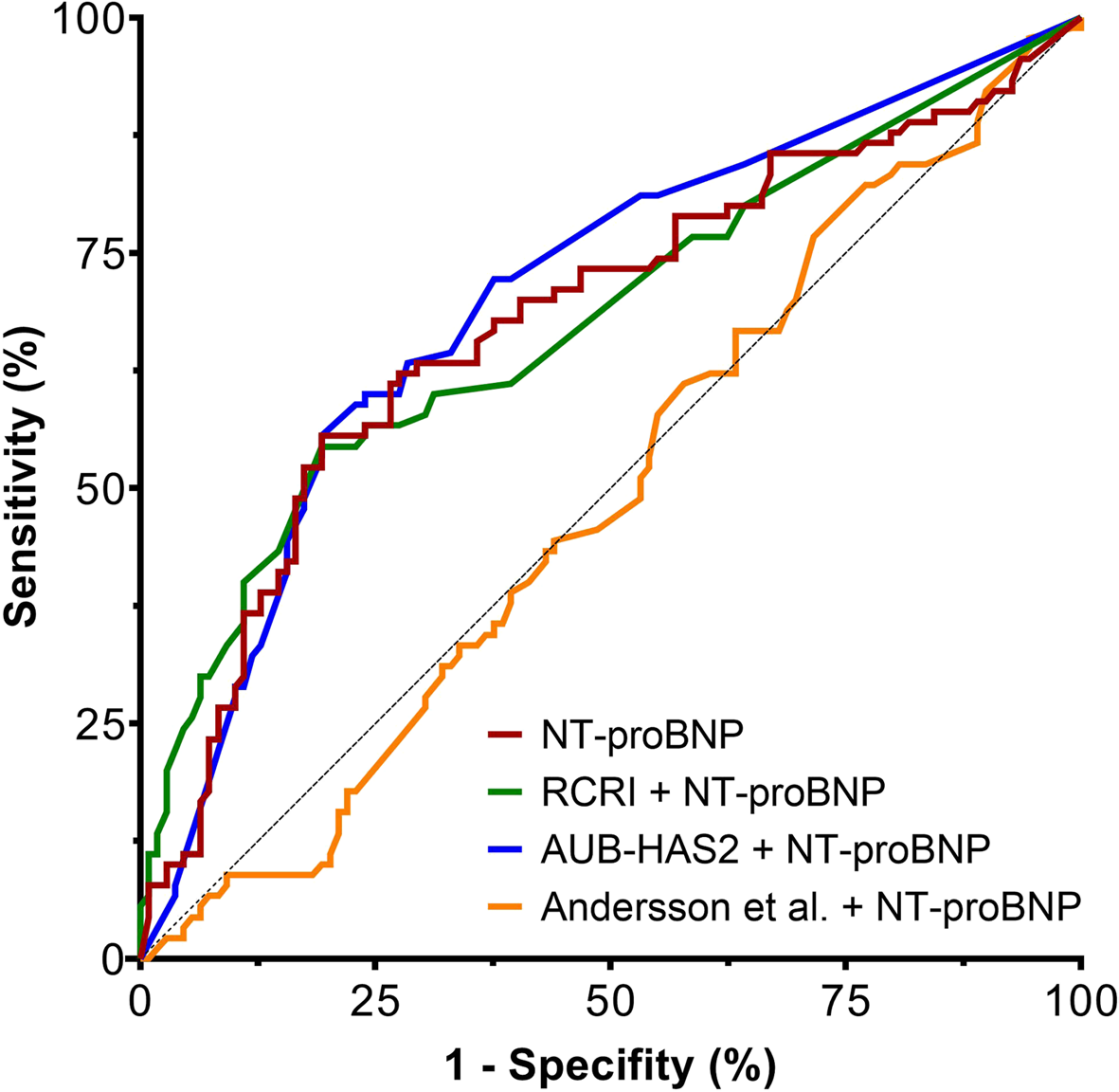
Predictive value of clinical risk scores combined with NT-proBNP. While predictivity of the revised cardiac risk Index (RCRI) and the American University of Beirut Cardiovascular Risk Index (AUB-HAS2) was improved, no improvement was realised using the score by Andersson et al.

Based on the multivariable logistic regression models, new scores were created using the original score components and the preoperative NT-proBNP cut-off. Each component’s influence was adjusted according to the respective multivariable model using its calculated odds ratio, with the components then summed to obtain the new scores. The formulas used to calculate these modified risk scores are shown in the *supplement*. Patients with preoperative NT-proBNP ≥ 450 pg/mL had higher optimised RCRI and AUB-HAS2 scores than those with preoperative NT-proBNP < 450 pg/mL (optimised RCRI: 1.05 [0.0–1.05] vs. 6.52 [5.41–7.69], optimised AUB-HAS2 0.6 [0.0–1.65] vs. 7.95 [6.90–8.55]; both *p* < 0.001), while their optimised Andersson scores remained comparable (5.64 [2.42–7.17] vs. 5.16 [2.42–7.14]; *p* = 0.552). While adding preoperative NT-proBNP and weighting the individual components by the multivariable model did not improve the predictive ability of the Andersson score for the CME, it did improve the predictive abilities of the RCRI and AUB-HAS2 (both *p* < 0.001; Table 3). The AUCROC for the optimised scores are shown in Fig. 3. However, while the numerically largest AUCROC was obtained by combining preoperative NT-proBNP with AUB-HAS2, the AUCROC for the modified RCRI and AUB-HAS2 scores did not differ significantly from that of preoperative NT-proBNP alone.

**Table 3:** The predictivity of the modified risk scores incorporating preoperative NT-proBNP

Score	AUCROC	*p*	Cut-off	Sensitivity	Specificity	*p* (NT-proBNP alone)	*p* (score alone)
Composite morbidity endpoint							
Optimised Revised Cardiac Risk Index	0.679	≤ *0.001*	4.693	54.4%	80.7%	0.988	< *0.001*
Optimised AUB-HAS2 risk score	0.703	< *0.001*	3.111	55.6%	80.7%	0.367	*0.001*
Optimised Andersson et al. risk score	0.492	0.845				*0.001*	0.901

Abbreviations: *AUCROC* area under the receiver operating characteristic curve, *AUBHAS2* American University of Beirut Cardiovascular Risk Index

## Discussion

The most important result of this exploratory analysis was its demonstration that the predictive ability of simple cardiac risk scores, such as the RCRI and AUB-HAS2, could be improved by adding the preoperative NT-proBNP cut-off.

Our data also showed that the score proposed by Andersson et al. is insufficient to assess postoperative morbidity, including all individual components of the CME in this study, after non-cardiac surgery in cohorts containing both patients with and without HF. The Andersson score has been validated for 30-day mortality risk in a large non-cardiac surgery cohort in which all patients had a known history of HF (Andersson et al. 2014). Therefore, the impact of the isolated Andersson score criteria appears to differ substantially from what is needed when assessing perioperative morbidity risk in mixed cohorts. This finding might be particularly aggravated by age and body mass index being weighted sevenfold and fourfold in the original score, respectively. When the Andersson score is calculated, the data from our cohort suggest that these two criteria are overemphasised. It is plausible that age and weight alone do not determine postoperative morbidity without other comorbidities in general populations. Therefore, its predictive ability to identify patients at risk for postoperative complications was non-existent in our analysis.

The correlation between preoperative NT-proBNP, which was predictive of postoperative complications in our cohort, and the Andersson score was very weak. The correlation was also weak between preoperative NT-proBNP and the RCRI, which also failed to significantly predict the CME in our small cohort. However, its predictive ability for AKI was sufficient and comparable to preoperative NT-proBNP. Therefore, we generated some preliminary evidence that the RCRI may also be used to predict postoperative AKI after major non-cardiac surgery. The RCRI has generally been shown to discriminate moderately well in mixed non-cardiac surgery cohorts but did not perform well in vascular surgery cohorts (Ford et al. 2010). However, it was shown that its predictivity for major adverse cardiovascular events could be improved by preoperative biomarkers, such as high-sensitivity cardiac troponin T/I and NT-proBNP (Kyeong et al. 2008; Vernooij et al. 2021; Rodseth et al. 2011; Park et al. 2011). Unfortunately, there is still a lack of clinically applicable strategies combining the RCRI with preoperative biomarker screening.

In our analysis, the modified RCRI, including the weighted preoperative NT-proBNP cut-off and original RCRI components, showed improved predictivity compared to the original RCRI. However, the multivariable logistic regression model showed that preoperative NT-proBNP had an almost fivefold greater adjusted predictive ability for the CME than the RCRI components. A meta-analysis by Rodseth et al. showed that reclassifying the RCRI using a preoperative NT-proBNP cut-off improved risk prediction for cardiovascular deaths and nonfatal myocardial infarction (Rodseth et al. 2011). Therefore, our data are consistent with previously published data, expanding the improvability of the RCRI to the field of postoperative morbidity. However, it must be noted that the original RCRI criteria had only a small impact on the modified score because of the strongly weighted preoperative NT-proBNP cut-off. Therefore, this finding once again underscores the superiority of preoperative NT-proBNP alone over clinical scores, such as the RCRI, in predicting the CME. In addition, its overall predictivity for the CME could not be improved compared to preoperative NT-proBNP alone.

The AUB-HAS2 showed a good correlation with preoperative NT-proBNP in our analysis. It also showed better risk prediction than the RCRI for the CME but for ADHF and postoperative infections. The criteria used in the AUB-HAS2 seem to be of higher relevance for the perioperative outcome than the parameters of the other scores, leading to the improved predictive value for the CME. For example, symptoms of heart disease are a criterion that can be positive, even if the patient has not yet diagnosed with HF or chronic coronary syndrome. Furthermore, anaemia is not considered in the RCRI and Andersson score; however, preoperative anaemia is strongly associated with postoperative AKI and adverse outcomes (Katayama et al. 2021). The AUB-HAS2 was initially validated for risk prediction of 30-day death, myocardial infarction, and stroke in a general non-cardiac surgery cohort (Dakik et al. 2019b). It was subsequently shown to be predictive in various surgical subpopulations, including vascular surgery (Dakik et al. 2019a; Msheik et al. 2021). Therefore, we expanded the evidence that AUB-HAS2 can also predict postoperative morbidity, showing better discriminatory power than the RCRI in our cohort. Our data is consistent with previous analyses showing that AUB-HAS2 was superior to the RCRI (Dakik et al. 2019a, 2019b). AUB-HAS2 predicted risk for the CME was comparable to that of preoperative NT-proBNP alone. alone. Therefore, both tools can be suitably used to assess perioperative morbidity risk in patients undergoing non-cardiac surgery. While the individual strength of the AUB-HAS2 depends on accurate information in the patient’s history, preoperative NT-proBNP measurement offers an additional independent parameter.

However, it must be noted that NT-proBNP can be negative in up to 20% of patients with HF with preserved ejection fraction and might be influenced by other factors, such as age, weight, sex, and renal function (McDonagh et al. 2021). Therefore, combining preoperative NT-proBNP and clinical risk scores appears attractive. When preoperative NT-proBNP was combined with the AUB-HAS2 after the multivariable analysis in our study, the AUCROC was numerically improved compared to AUB-HAS2 or preoperative NT-proBNP alone. However, the absolute difference in the AUCROC was small, showing statistical significance to the original score but not NT-proBNP alone, potentially reflecting our study’s small sample size. Furthermore, preoperative NT-proBNP was weighted stronger than all other AUB-HAS2 parameters together. Therefore, patients with preoperative NT-proBNP below the cut-off but with clinically relevant chronic HF would be at risk of false-negative classification with the modified score calculated in our study. These findings suggest that risk stratification should still be performed by combining biomarkers and risk scores derived from the patient’s history because no existing clinical risk scores sufficiently include preoperative biomarkers.

Nevertheless, our data showed that combining preoperative NT-proBNP with established risk scores might improve outcome prediction for postoperative morbidity. Furthermore, while the AUB-HAS2 was validated for death, myocardial infarction, and stroke at 30 days, it could also predict the morbidity measures analysed in our study. These morbidity events occur more frequently than the fatal outcomes initially assessed. For example, the AUB-HAS2 was validated in a cohort with a 1.2% rate of major adverse cardiovascular events (Dakik et al. 2019b). However, there are good reasons to consider morbidity measures, such as those analysed in our study, when assessing perioperative risk. Postoperative morbidity after non-cardiac surgery is not only individually relevant but is also economically important because the morbidity events analysed in our study have already caused longer hospital and intensive care unit stays (Schmidt et al. 2024). Therefore, when risk scores and biomarker screening jointly indicate high perioperative risk, distinct therapeutic strategies are indicated and should be evaluated in further studies.

Our study had several limitations that must be acknowledged. First, its sample size was small, and the initial sample size was not powered to assess differences in perioperative risk scores. Therefore, the results of this pilot analysis should be interpreted as hypothesis-generating because its small sample size limited its statistical analysis. For example, advanced statistical measures, such as machine learning models, could not be implemented in our study to improve predictivity for the CME. Second, we did not validate the modified scores in an independent validation cohort. Further studies with larger cohorts are necessary to create more accurate predictive models considering preoperative NT-proBNP testing after major non-cardiac surgery. Third, our study only considered elective non-cardiac and non-vascular surgeries. Therefore, the predictive abilities of the analysed scores were impaired because not all of their criteria were covered by this analysis. For example, the AUB-HAS2 considers both vascular and emergency surgery for risk prediction; however, it still had the best predictivity of all analysed scores in our cohort. Fourth, only patients undergoing surgery with intermediate or high surgical risk were analysed. Therefore, our study did not examine risk prediction in patients with low surgical risk but potentially high patient-related risk. The predictivity of the analysed scores may be reduced in patients undergoing low-risk surgery, which could be classified as at high risk for the CME.

## Conclusions

Our secondary analysis of a single-centre observational trial showed that the predictivity of postoperative morbidity varies markedly between the available simple perioperative risk scores. Our data revealed that combining preoperative NT-proBNP with clinical risk scores could generally enhance predictivity for postoperative morbidity compared to the clinical risk scores alone, as shown for the RCRI. However, the predictive ability of preoperative NT-proBNP alone was comparable to that of the AUB-HAS2. Therefore, adding only preoperative NT-proBNP to established risk scores may be insufficient to improve the risk stratification of patients undergoing major non-cardiac surgery. New scores that include preoperative biomarkers should be evaluated in large multicentre cohorts to evaluate which clinical parameters should be added to maximise their predictivity.

### Supplementary Information

Supplementary Material 1: Supplementary figure: Fig. 1. Scatter plots of preoperative NT-proBNP and clinical risk scores. Spearman’s rank correlation coefficients (rs) indicate that preoperative NT-proBNP is A weakly correlated with the revised cardiac risk index (RCRI) but B strongly correlated with the American University of Beirut Cardiovascular Risk Index (AUB-HAS2). C Preoperative NT-proBNP is only very weakly correlated with the Andersson score.

## Data Availability

The datasets used and/or analysed during the current study are available from the corresponding author on reasonable request.

## Abbreviations

ADHF: Acute decompensated heart failure
AKI: Acute kidney injury
AUB-HAS2: American University of Beirut Cardiovascular Risk Index
AUCROC: Area under the receiver operating characteristic curve
CME: Composite morbidity endpoint
ESC: European Society of Cardiology
HF: Heart failure
NT-proBNP: N-terminal prohormone of BNP
POD: Postoperative day
RCRI: Revised cardiac risk index

## References

[CR1] Andersson C, Gislason GH, Hlatky MA, Søndergaard KB, Pallisgaard J, Smith JG (2014). A risk score for predicting 30-day mortality in heart failure patients undergoing non-cardiac surgery. Eur J Heart Fail.

[CR2] Dakik HA, Chehab O, Eldirani M, Sbeity E, Karam C, Abou Hassan O (2019). Aub-has2 cardiovascular risk index: performance in surgical subpopulations and comparison to the Revised Cardiac Risk Index. J Am Coll Cardiol.

[CR3] Dakik HA, Chehab O, Eldirani M, Sbeity E, Karam C, Abou Hassan O (2019). A new index for pre-operative cardiovascular evaluation. J Am Coll Cardiol.

[CR4] Farzi S, Stojakovic T, Marko T, Sankin C, Rehak P, Gumpert R (2013). Role of N-terminal pro B-type natriuretic peptide in identifying patients at high risk for adverse outcome after emergent non-cardiac surgery. Br J Anaesth.

[CR5] Ford MK, Beattie WS, Wijeysundera DN. Systematic review: prediction of perioperative cardiac complications and mortality by the Revised Cardiac Risk Index. Ann. Intern. Med. 2010. p. 26–35. 10.7326/0003-4819-152-1-201001050-00007. 10.7326/0003-4819-152-1-201001050-00007 20048269

[CR6] Gerber Y, Weston SA, Redfield MM, Chamberlain AM, Manemann SM, Jiang R (2015). A contemporary appraisal of the heart failure epidemic in Olmsted County, Minnesota, 2000 to 2010. JAMA Intern Med.

[CR7] Groenewegen A, Rutten FH, Mosterd A, Hoes AW (2020). Epidemiology of heart failure. Eur J Heart Fail.

[CR8] Gualandro DM, Puelacher C, Chew MS, Andersson H, Lurati Buse G, Glarner N (2023). Acute heart failure after non-cardiac surgery: incidence, phenotypes, determinants and outcomes. Eur J Heart Fail.

[CR9] Halvorsen S, Mehilli J, Cassese S, Hall TS, Abdelhamid M, Barbato E, et al. 2022 ESC guidelines on cardiovascular assessment and management of patients undergoing non-cardiac surgery. Eur Heart J. 2022; doi:10.1093/eurheartj/ehac270. 10.1093/eurheartj/ehac270 36017553

[CR10] Katayama HT, Gomes BC, Lobo SMA, Chaves RCF, Corrêa TD, Assunção MSC (2021). The effects of acute kidney injury in a multicenter cohort of high-risk surgical patients. Ren Fail.

[CR11] Kyeong HY, Myung HJ, Seok KO, Choi JH, Sang JR, Eun MP (2008). Preoperative plasma N-terminal pro-brain natriuretic peptide concentration and perioperative cardiovascular risk in elderly patients. Circ J.

[CR12] Lee TH, Marcantonio ER, Mangione CM, Thomas EJ, Polanczyk CA, Cook EF (1999). Derivation and prospective validation of a simple index for prediction of cardiac risk of major noncardiac surgery. Circulation.

[CR13] McDonagh TA, Metra M, Adamo M, Gardner RS, Baumbach A, Böhm M (2021). 2021 ESC guidelines for the diagnosis and treatment of acute and chronic heart failure. Eur Heart J.

[CR14] Msheik A, Kaspar C, Mailhac A, Hoballah JJ, Tamim H, Dakik HA (2021). Performance of the AUB-HAS2 cardiovascular risk index in vascular surgery patients. Vasc Med (united Kingdom).

[CR15] Park SJ, Choi JH, Cho SJ, Chang SA, Choi JO, Lee SC (2011). Comparison of transthoracic echocardiography with N-terminal pro-brain natriuretic peptide as a tool for risk stratification of patients undergoing major noncardiac surgery. Korean Circ J.

[CR16] Rodseth RN, Lurati Buse GA, Bolliger D, Burkhart CS, Cuthbertson BH, Gibson SC (2011). The predictive ability of pre-operative B-type natriuretic peptide in vascular patients for major adverse cardiac events: an individual patient data meta-analysis. J Am Coll Cardiol.

[CR17] Schmidt G, Frieling N, Schneck E, Habicher M, Koch C, Rubarth K, et al. Preoperative routine measurement of NT-proBNP predicts postoperative morbidity after non-cardiac surgery with intermediate or high surgical risk: an observational study. BMC Anesthesiol. 2024;24. 10.1186/s12871-024-02488-8. 10.1186/s12871-024-02488-8 PMC10960410 38521898

[CR18] Shah KS, Xu H, Matsouaka RA, Bhatt DL, Heidenreich PA, Hernandez AF (2017). Heart failure with preserved, borderline, and reduced ejection fraction: 5-year outcomes. J Am Coll Cardiol.

[CR19] Vernooij LM, van Klei WA, Moons KGM, Takada T, van Waes J, Damen JAAG (2021). The comparative and added prognostic value of biomarkers to the revised cardiac risk index for preoperative prediction of major adverse cardiac events and all-cause mortality in patients who undergo noncardiac surgery. Cochrane Database Syst Rev.

[CR20] Walther CP, Podoll AS, Finkel KW (1995). Summary of clinical practice guidelines for acute kidney injury. Hosp Pract.

