# Supplementary material for: Comparison of preoperative NT-proBNP and simple cardiac risk scores for predicting postoperative morbidity after non-cardiac surgery with intermediate or high surgical risk
Source: Perioper Med (Lond). 2024 May 17;13:44. doi: 10.1186/s13741-024-00400-z (PMC11100121; doi:10.1186/s13741-024-00400-z)
Supplement: Supplementary file 1 — Supplementary Material 1: Supplementary figure: Fig. 1. Scatter plots of preoperative NT-proBNP and clinical risk scores. Spearman’s rank correlation coefficients (rs) indicate that preoperative NT-proBNP is A weakly correlated with the revised cardiac risk index (RCRI) but B strongly correlated with the American University of Beirut Cardiovascular Risk Index (AUB-HAS2). C Preoperative NT-proBNP is only very weakly correlated with the Andersson score. [file 13741_2024_400_MOESM1_ESM.docx]

**Supplement.**

**
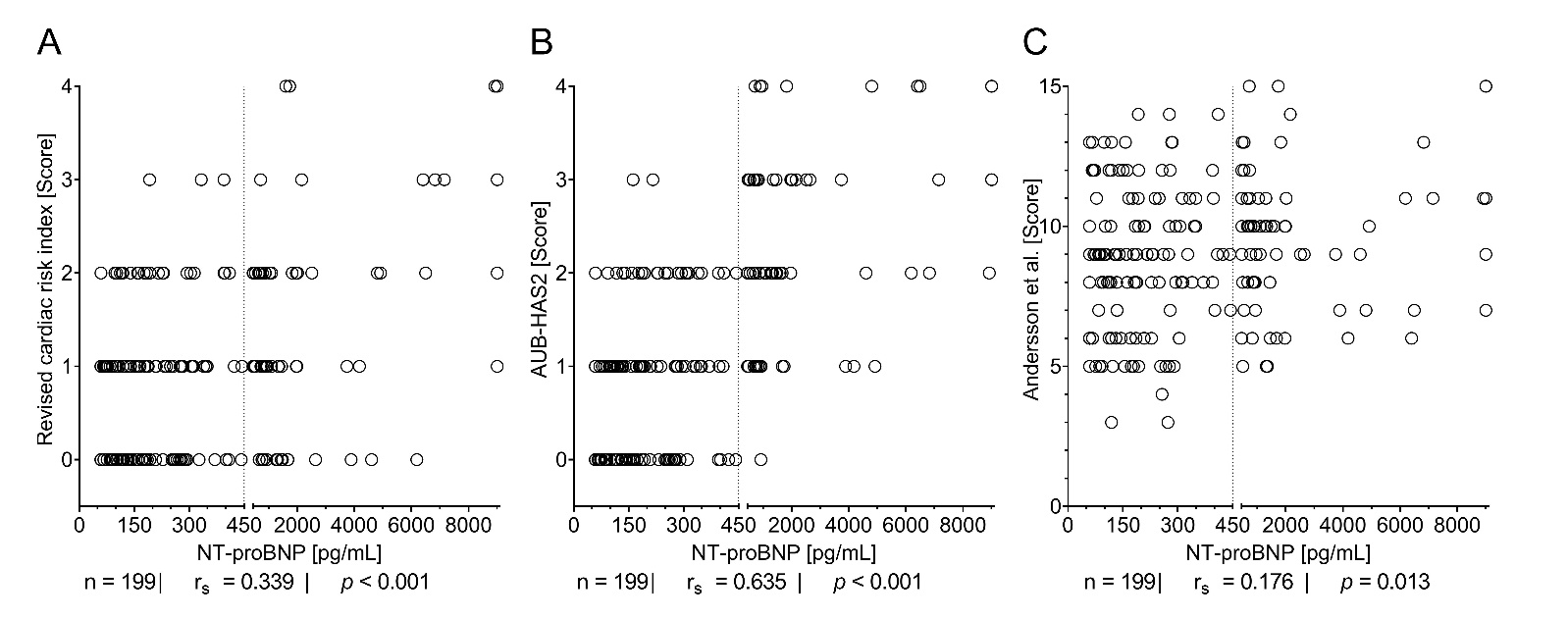
Figure 1.** Scatter plots of preoperative NT-proBNP and clinical risk scores. Spearman’s rank correlation coefficients (r_s_) indicate that preoperative NT-proBNP is **A** weakly correlated with the revised cardiac risk index (RCRI) but **B** strongly correlated with the American University of Beirut Cardiovascular Risk Index (AUB-HAS2). **C** Preoperative NT-proBNP is only very weakly correlated with the Andersson score.

Optimised risk scores were calculated according to the following formulas. Each parameter was assigned a value of 1 if present, and 0 if absent.

$Optimised revised cardiac risk index=$

$$\left( coronary artery disease \times0.47 \right)$$

$$+\left( chronic heart failure\times1.70 \right)$$

$$+\left( insulin therapy\times1.64 \right)$$

$$+\left( cerebrovascular disease\times1.05 \right)$$

$$+\left( high-risk type of surgery\times1.05 \right)$$

$$+\left( serum creatinine>2mg / dL\times1.75 \right)$$

$$+(NT-proBNP>450 pg/mL \times4.94)$$

$Optimised AUB-HAS2 risk score=$

$$\left( angina or dyspnea \times1.01 \right)$$

$$+\left( history of heart disease\times0.60 \right)$$

$$+\left( haemoglobin<12g/dL\times1.65 \right)$$

$$+\left( age>75years\times1.16 \right)$$

$$+(NT-proBNP>450 pg/mL \times5.14)$$

$Optimised Andersson et al. risk score=$

$$\left( insulin therapy \times1.53 \right)$$

$$+\left( cerebrovascular disease\times1.56 \right)$$

$$+\left( renal disease\times0.96 \right)$$

$$+\left( high-risk type of surgery\times1.06 \right)$$

$$+\left( overweight \times0.71 \right)$$

$$+\left( normal weight\times0.76 \right)$$

$$+\left( underweight\times0.69 \right)$$

$$+\left( age 66-75 years\times0.74 \right)$$

$$+\left( age 76-85 years\times1.04 \right)$$

$$+\left( age\geq85years\times1.03 \right)$$

$$+\left( male\times0.75 \right)$$

$$+(NT-proBNP>450 pg/mL \times4.41)$$
